# Airway management and ventilation techniques in resuscitation during advanced life support: an update

**DOI:** 10.1186/s44158-024-00195-x

**Published:** 2024-08-24

**Authors:** Clemens Kill, Randi Katrin Manegold, David Fistera, Joachim Risse

**Affiliations:** 1 Center of Emergency Medicine, University Hospital Essen, Essen, D-45147 Germany; 2 Center of Emergency Medicine, University Hospital Essen, Hufelandstrasse 55, Essen, 45122 Germany

**Keywords:** Advanced life support (ALS), Cardiopulmonary resuscitation (CPR), Airway management, Ventilation modes, Chest compression synchronized ventilation (CCSV), Best ventilation strategy for CPR

## Abstract

For many years, ventilation has been an essential part of advanced life support (ALS) in cardiopulmonary resuscitation (CPR). Nevertheless, there is little evidence about the best method of ventilation during resuscitation for both out-of-hospital cardiac arrest (OHCA) and inhospital cardiac arrest (IHCA) patients. Effective ventilation is one of the two main keys to successful resuscitation. In this context, the question always arises as to which airway management, along with which ventilation mode, constitutes the best strategy. Conventional ventilation modes are not designed for cardiac arrest and show important limitations that must be considered when used in CPR. Manual ventilation without the use of an automated transport ventilator (ATV) could be shown to be uncontrolled in applied volumes and pressures and should be avoided. Mechanical ventilation with an ATV is therefore superior to manual ventilation, but both volume- and pressure-controlled ventilation modes are significantly influenced by chest compressions. With the newly designed chest compression synchronized ventilation (CCSV), a special ventilation mode for resuscitation is available. Further research should be conducted to obtain more evidence of the effect of ventilation during CPR on outcomes following OHCA and not only about how to secure the airway for ventilation during CPR.

## Background

For more than half a century, “CPR” described the procedures of cardiopulmonary resuscitation. The CPR procedure, therefore, consists of both parts cardiac and pulmonary resuscitation. Although many research efforts have been undertaken with a focus on the heart to achieve the restoration of spontaneous circulation, only limited research data are available concerning “pulmonary” resuscitation [1, 2]. Although ventilation during advanced life support has been recommended for decades, there is little evidence about the best method of ventilation. Due to a lack of research, there have also been few technical advancements that adequately considered the specific pathophysiology during resuscitation. The primary focus of research was more on understanding the relationship and determining which airway management technique for ventilation provides the best outcome [3, 4]. The adoption of groundbreaking and scientifically grounded ventilation strategies for resuscitation is still lacking. Nevertheless, rescuers worldwide use ventilation via many different techniques that are often driven by personal experience and opinions [5]. This narrative review is divided into airway management to apply the ventilation techniques and the ventilation techniques themselves and describes the mechanisms of ventilation during resuscitation and provides existing data about possible ventilation strategies.

## Main text

### How much oxygen is needed during CPR?

During CPR with chest compressions, the perfusion of the body is markedly reduced; therefore, oxygen delivery to both the brain and heart may also be critically low [6]. In contrast to the negative effects of hyperoxygenation in the early postresuscitation period after the return of spontaneous circulation, there is some evidence supporting higher oxygen partial pressures during CPR [7–9]. Based on the reduced perfusion of the lungs during CPR as well as the dystelectasis and atelectasis caused by chest compressions [10], the oxygenation of the blood is decreased, and PaO2 values are lower than those associated with ventilation in living patients.

**Fig. 1:**
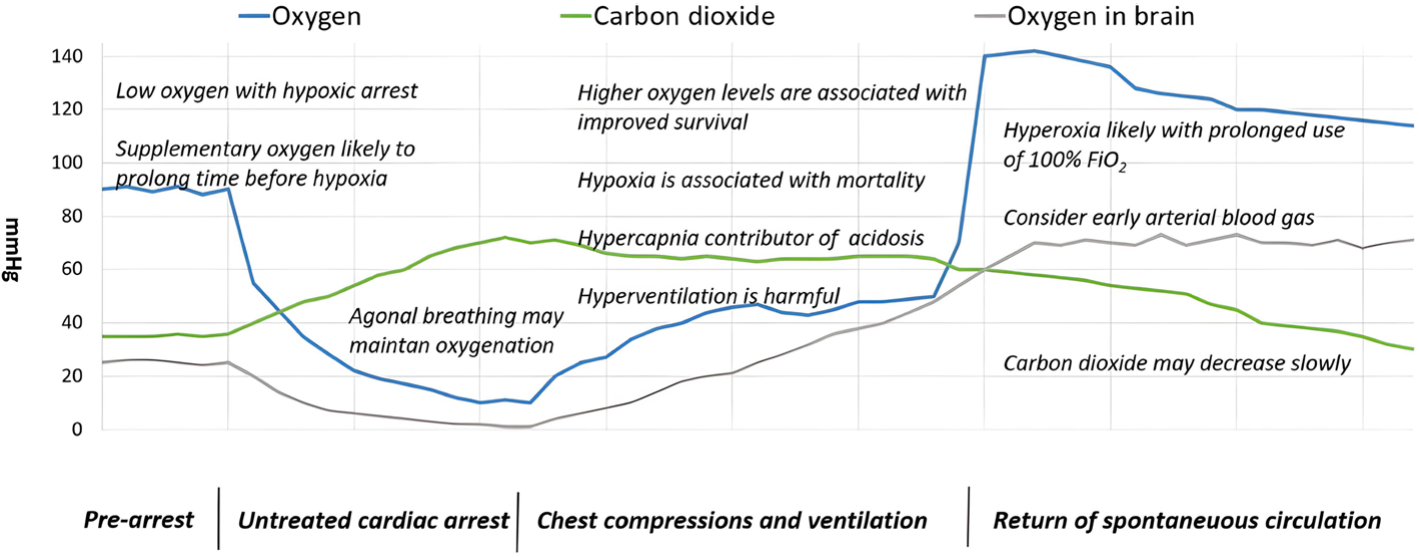
Changes in oxygenation and decarboxylation during resuscitation. From: M. B. Skrifvars, T. M. Olasveengen, and Giuseppe Ristagno. Intensive Care Med (2019) 45:284–286

For these reasons, the use of a maximum inspiratory oxygen concentration for ventilation of adults in cardiac arrest is still widely accepted and recommended [11–13]. Figure 1 shows an overview of the changes in oxygen and carbon dioxide levels during the phases of cardiac arrest [14].

### How much ventilation is necessary during CPR?

For many years, international guidelines on resuscitation recommend ventilation with pure oxygen using a respiratory rate of 10 breaths per minute, but no further recommendations concerning tidal volumes and ventilation modes are mentioned in the resuscitation guidelines [15]. This recommendation is based on data on the negative effects of hyperventilation [16]. Further recommendations about adjusting ventilation during prolonged resuscitation do not exist, although we know that the recommended ventilation volumes are often not sufficient to avoid respiratory acidosis [17]. However, hypoventilation has also been shown to be associated with worse outcomes even in the early stage of experimental resuscitation [18]. Currently, there is a lack of evidence on how to adjust ventilation once arterial blood gas analysis is available during CPR because we do not know in detail the effects of both hypocapnia and hypercapnia on cerebral perfusion under the special circumstances of cardiac arrest. Therefore, many clinicians try to avoid excessive hypercapnia and respiratory acidosis by increasing ventilation volumes.

### What is different in ventilation during resuscitation?

During cardiopulmonary resuscitation, there are different circumstances for ventilation in contrast to the ventilation of patients with an intact cardiovascular system. The first and most important issue is the nonbeating heart. To maintain minimal perfusion of the lungs and all vital organs during resuscitation, chest compressions are performed. The supposed working principle of chest compressions is a combination of direct compression of the heart and a cyclic change in intrathoracic pressure. By both compressing the heart and vessels to maintain blood flow and pressure, a forward-directed blood flow is generated.

The cardiac output generated by chest compressions is quite low, leading to only minimal circulation, and thus, pulmonary perfusion is reduced compromising effective gas exchange. Chest compressions also cause changes in lung volume by compressing lung tissue during each chest compression.

In respiratory medicine, we typically differentiate between the controlled ventilation of apneic patients and the assisted ventilation of those with insufficient spontaneous breathing. In controlled ventilation modes, all actions of the ventilator are defined by the operator, and the patient does not trigger the ventilator at all; therefore, pressures, volumes, and flows are influenced only by the resistance and compliance of the lungs.

For patients with inadequate spontaneous breathing, assisted ventilation enables ventilator support through a ventilator triggered by spontaneous respiratory effort.

Most patients are apneic in cardiac arrest, but each chest compression causes a small expiratory and inspiratory gas flow synchronous with each chest compression that interacts with the functions of any ventilator. These small in-expiratory tidal volumes override the settings of both volume- and pressure-controlled ventilation modes and may critically influence both ventilation and ventilator function, as described in the section on mechanical ventilation [19, 20].

Depending on the level of airway management (none, extraglottic airway device EGA, or endotracheal intubation ETI) during resuscitation, there are several options for ventilation:

1. Passive ventilation
2. Manual bag-mask ventilation
3. Manual bag-EGA ventilation
4. Manual bag-ETI ventilation
5. Mechanical EGA ventilation
6. Mechanical ETI ventilation

All six procedures have a justified impact on ventilation during CPR and are described individually below. However, the main focus of this review is on the various potential ventilation modes used during advanced life support (ALS) for airways secured by ETI.

#### Passive ventilation

Passive ventilation is considered the lowest level of airway management during resuscitation. With open airways and occasional gasping, as well as through the passive recoil forces of the chest, a certain amount of gas exchange can occur during chest compressions [21]. This is particularly helpful in the first minutes of resuscitation. With an extended duration of resuscitation, this form of ventilation becomes inadequate. In the case of asphyxial cardiac arrest, this type of ventilation is not sufficient.

#### Manual bag-mask ventilation

Manual bag mask ventilation is typically performed by all professional rescuers during resuscitation until ventilation is secured through ETI or advanced airway management (EGA). For individuals providing both chest compressions and ventilation, the compression-ventilation ratio is typically 30:2. After 30 compressions, 2 ventilations were provided. This ratio of 30:2 is anchored in the current basic life support and advanced life support guidelines to ensure sufficient ventilation during resuscitation in the absence of a secured airway, providing adequate tidal volume and avoiding gastric insufflation. However, even among skilled rescuers, this ratio is not always adhered to, and hyperventilation often occurs during cardiopulmonary resuscitation. Therefore, bag-mask ventilation is often an initial intervention. Nevertheless, if advanced airway management (such as an endotracheal tube or extraglottic airway device) is established, ventilation may be provided through that device. Once an endotracheal tube or extraglottic airway device is in place, this strict ratio is abandoned, and ventilation can be performed concurrently with chest compressions. Even in an experimental setting, it can be demonstrated that the use of ventilators to avoid excessive ventilation during CPR is applicable [22].

Limitations of bag-mask ventilation are airway obstruction, failure to achieve chest wall movement, and the increased risk of aspiration. The danger of pure bag-mask ventilation is always the increased risk of gastric insufflation, leading to subsequent regurgitation and aspiration. Due to a significant decrease in lower esophageal sphincter pressure and lung compliance at the onset of cardiac arrest, the risk is consistently elevated [23]. In a recent large study including 649,359 patients, bag-mask ventilation was associated with better outcome than patients receiving advanced airway management with EGA or ETI. However, these results are limited by the very short period of only 6 min from start CPR to return of spontaneous circulation (ROSC) in the bag-mask group compared to 14 min in the advanced airway group. Furthermore, the overall outcome in this nationwide Japanese study was only 2.2% for neurologically intact survival [24]. In another large retrospective study, ETI within the first 15 min of CPR was associated with lower favorable outcome compared to bag-mask ventilation, although it is not known if ETI itself might be disadvantageous or ETI was used because of difficulties in bag-mask ventilation as a rescue strategy [25].

#### Manual bag-EGA ventilation/mechanical EGA ventilation

The establishment of an EGA for ventilation in cardiopulmonary resuscitation is part of advanced airway management during ALS and is always the responsibility of trained medical personnel. Research has not proven whether ventilation via EGA or endotracheal intubation is ultimately the gold standard for airway management during cardiopulmonary resuscitation. On the one hand, patients who were provided with an EGA had a lower likelihood of achieving ROSC, a lower 30-day hospital discharge rate, and a poorer neurological treatment outcome than patients who are receiving mask ventilation without EGA or an endotracheal tube [26]. However, contradictory findings have been demonstrated in a recent prospective study [4].

Even with manual bag-EGA ventilation, as with manual bag-ETI ventilation, as shown below, there is a high risk of potentially uncontrolled excessive ventilation during resuscitation.

For experienced practitioners, ETI appears to be the gold standard and, therefore, the recommended form of airway control for the European Resuscitation Council (ERC) [11, 12].

A recent retrospective study indicated that patients who underwent resuscitation with ETI upon arrival at the clinic had significantly better ventilation parameters than those with a extraglottic airway (EGA). Patients with EGA exhibited overall poorer blood gas values and less frequently met the criteria for extracorporeal cardiopulmonary resuscitation (eCPR) than patients in whom airway management during resuscitation was performed using endotracheal intubation [27]. Airway management with ETI and ventilation through ETI appears to be advantageous, at least in the context of prolonged CPR. Patients with nonsynchronized mechanical chest compressions or transport under CPR likely benefit from ETI. This can be explained by the disadvantages of airway management during resuscitation using EGA, such as poorer oxygenation and ventilation conditions, which accumulate with prolonged resuscitation duration. However, these disadvantages with EGA do not have a significant impact initially or over a few minutes. The general superiority of this approach cannot be conclusively inferred at this time because of the retrospective study design. Further, prospective studies are needed. However, this can be regarded as a significant indication of the superiority of ETI, at least in the context of prolonged CPR. Therefore, a cautious recommendation for airway management via ETI should be considered. Based on this assumption, the subsequent part of the review predominantly focuses on ventilation during ALS and the potential ventilation modes with an airway secured by ETI.

#### Manual bag-ETI ventilation

Bag-to-ETI ventilation is most common in out-of-hospital cardiac arrest (OHCA) patients worldwide. It is easy to perform, no automated mechanical ventilator is needed on the scene, and many rescuers believe that it is important to “feel” ventilation during resuscitation. Some also argue that this allows them to better adopt ventilation for chest compression and may avoid excessively high inspiratory pressure. Unfortunately, these “feelings” lack evidence, and there are studies proving the opposite [28]. Manual ventilation can often be excessive or uncontrolled if no additional monitoring devices are used [29]. Additionally, manual ventilation requires the personal resources of qualified rescuers [30]. Based on these scientific data, there is no reason to use manual ventilation during ALS in OHCA patients or in the intrahospital cardiac arrest (IHCA) patients if mechanical ventilation is available.

#### Mechanical ETI ventilation

Today, automated transport ventilators (ATVs) are lightweight, mostly electronically controlled, and are often able to provide most ventilation modes used by intensive care unit (ICU) ventilators. An increasing number of ambulances worldwide are equipped with ventilators so that mechanical ventilation can be used not only IHCA but also for OHCA in the prehospital setting. There are numerous ventilation modes for both controlled and assisted ventilation. Most ventilation modes, however, have not been specifically developed for situations involving cardiopulmonary resuscitation and interactions with chest compressions.

### Different airway strategies for IHCA/OHCA patients

Cardiac arrest can occur in IHCA and OHCA. The differences in the various airway management strategies are largely determined by varying access to medical resources. IHCA occurs within a healthcare facility, where medical resources and staff are 24/7 available. One of the differences is the faster access to advanced airway equipment, such as ETI and EGA. Endotracheal intubation is often a common choice for airway management in IHCA patients. Extraglottic airway devices (e.g., laryngeal mask airways) may also be used, especially in cases where intubation is challenging or delayed. However, the key difference is the availability of highly trained medical staff with increased expertise in advanced airway management compared to OHCA patients. A higher skill level of providers might be crucial. In contrast, with OHCA, this resource and immediate access to advanced airway equipment may be limited. Therefore, the initial emphasis in OHCA is more on high-quality bystander CPR and early defibrillation. A long period of passive ventilation and bag-mask ventilation by emergency medical service (EMS) providers is common before the establishment of advanced airways. Furthermore, due to lower training requirements, EMS providers often opt for EGA devices and do not necessarily perform ETI as a first-line intervention during resuscitation. This is because intubation may be challenging in the field due to various factors, such as suboptimal patient positioning, limited lighting, and the absence of skilled personnel.

Therefore, in OHCA patients, extraglottic airway devices may be preferred over endotracheal intubation in these situations. In conclusion, airway management may be more difficult in OHCA settings than in IHCA settings due to limited resources and difficult outdoor conditions.

However, the current literature indicates that IHCA patients, despite purportedly experiencing initially faster and better resuscitation conditions within the hospital, currently do not exhibit a significant survival advantage over patients who have an OHCA [31, 32]. According to a recent multivariate analysis, the location of cardiac arrest, whether IHCA or OHCA, was not an independent predictor of functional outcome [33]. Ultimately, as with OHCA, it remains unclear which airway strategy is optimal for advanced airway management during an IHCA. To better understand this, in addition to the existing studies on OHCA, there is a need for future randomized control trials, such as the AIRWAYS-3 study, for IHCA [34]. The key to airway management during resuscitation might be to balance the need for prompt intervention with either EGA or ETI with the available expertise and equipment in each specific scenario, whether OHCA or IHCA.

In all studies on advanced airway management during resuscitation, there is a consistent limitation in comparability attributed to variations in country-specific EMS systems, particularly in the context of OHCA.

The availability of modern airway equipment for the advanced airway management of various EMS systems plays an important role. The range of instruments available for advanced airway management may vary, including access to endotracheal intubation devices, extraglottic airway devices, videolaryngoscopy, and other related devices.

The differences between individual EMS systems in airway management during CPR might be caused by the consistent use and adherence to standard operating procedure (SOP) protocols and guidelines as well as their integration into the broader healthcare system of the respective country.

Finally, cultural and ethical considerations always play a role in shaping the approach to advanced airway management. These may include considerations related to consent, withholding, or withdrawing interventions and end-of-life care.

### Interaction between chest compressions and ventilation

Ventilation of patients without cardiac arrest may be performed either controlled or in an assisted mode triggered by the patient’s efforts to breathe. Even small in- and expiratory gas flows that can occur during insufficient respiration are detected by the ventilator to support the patient. In cardiac arrest, there is—except for an initial period of agonal respiration during the first minutes of cardiac arrest—an apneic patient. When chest compressions are performed and the airway is secured, there is cyclic in- and expiratory gas flow synchronous to each chest compression. Each compression leads to a small expiratory gas flow, and each decompression leads to an inspiratory gas flow. These tidal volumes are only small and less than 1 ml/kg body weight and seem to not be sufficient for gas exchange during longer resuscitation efforts [20].

When using standard ventilation modes designed for the controlled or assisted ventilation of living patients, the described cyclic in- and expiratory gas flows might interfere with the ventilator’s function. The cyclic in- and expiratory gas flows caused by chest compressions may be detected as spontaneous breathing efforts by the ventilator leading to uncontrolled ventilator support. During each single chest compression, the lung volume is significantly reduced, leading to cyclic changes in lung compliance corresponding to the chest compression rate. Therefore, ventilation parameters might strongly differ from preset values during chest compressions. Speer et al. showed that during chest compressions, the preset tidal volumes in the volume-controlled ventilation mode, intermittent positive pressure ventilation (IPPV), are too low, and the peak inspiratory pressures in the pressure-controlled ventilation mode, biphasic positive airway pressure (BIPAP), exceed the preset values [35].

### Intermittent positive pressure ventilation and biphasic positive airway pressure ventilation during CPR

**Fig. 2:**
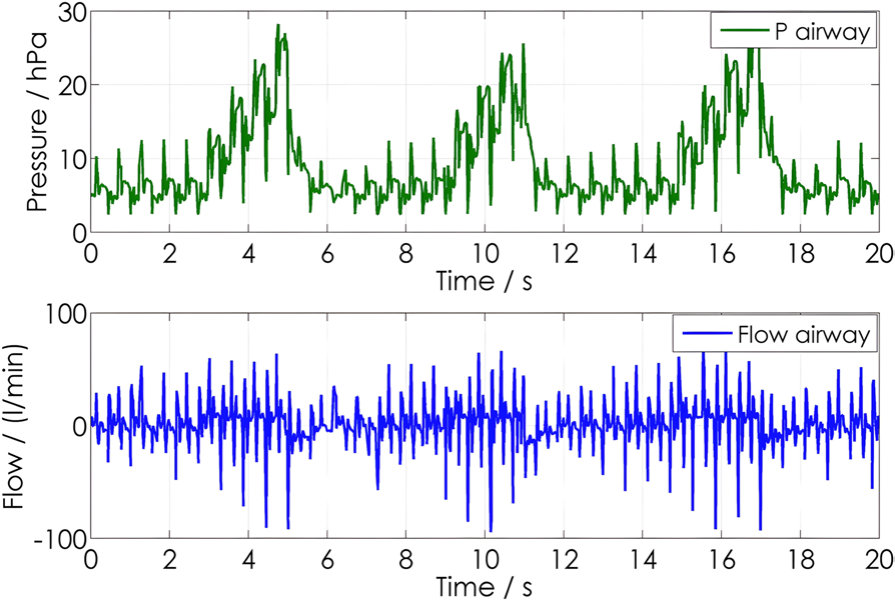
Pressure and flow curve of IPPV with chest compressions 100/min. IPPV parameter: (Tv 7 ml/kg, *f* = 10/min, no PEEP). From Neuhaus C. et al.: Mechanical ventilation during CPR: Influence of intermitted positive pressure ventilation and bilevel ventilation on tidal volumes in a pig model. Poster presentation on ERC Congress Porto 2010, 2*–*4th Dec 2010, cited in: 10.1016/j.resuscitation.2010.09.259

When using ventilation with volume-controlled ventilation mode during ALS, there are two major issues to consider. The preset tidal volume will be lower than previously defined depending on settings for max pressure, and the measurement of expiratory tidal volumes will be disturbed by chest compression-induced gas flows [22] (Fig. 2). Depending on the type of ventilator, especially in ATVs, it might also happen that during expiration, the inspiratory valves are closed, and inspiratory gas flow caused by chest compressions may lead to the opening of an emergency valve to room air [36].

**Fig. 3:**
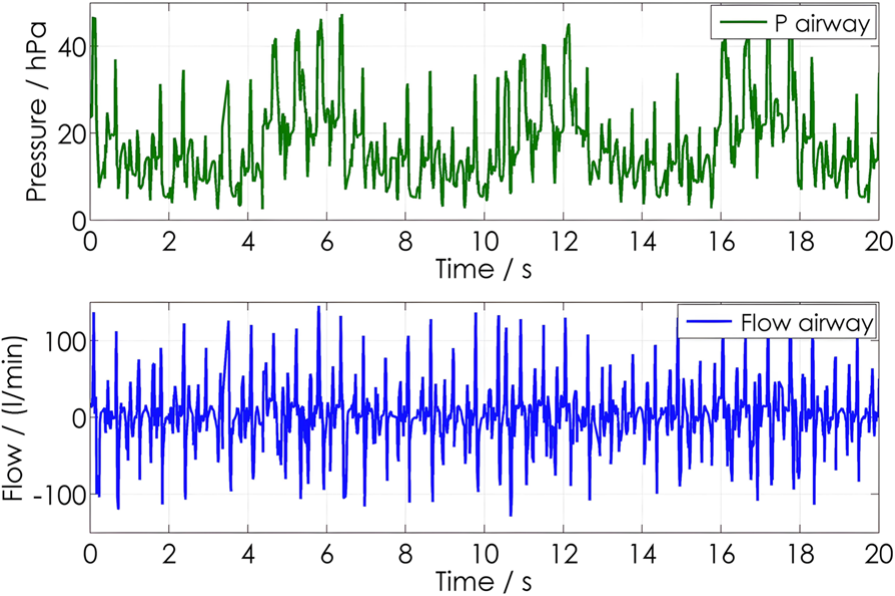
Pressure and flow curves of BIPAP with chest compressions (100/min) BIPAP (pinsp 15–19 mbar to achieve a Tv of 7 ml/kg, *f* = 10/min, *PEEP* = 5 mbar). From Neuhaus C. et al.: Mechanical ventilation during CPR: influence of intermitted positive pressure ventilation and bilevel ventilation on tidal volumes in a pig model. Poster presentation on ERC Congress Porto 2010, 2–4th Dec 2010, cited in: 10.1016/j.resuscitation.2010.09.259

Ventilation with pressure-controlled modus during ALS allows cyclic in- and expiratory gas to flow both at the inspiratory and expiratory pressure levels (Fig. 3). Experimental data from an animal study using pigs demonstrated advantages over volume-controlled ventilation mode during the cardiac arrest and post-resuscitation periods, including reduced pulmonary shunt immediately after ROSC, a tendency towards lower inspiratory pressures, and decreased expression of tumor necrosis factor alpha in hippocampal tissue [37]. These results were supported by prehospital human data comparing the IPPV, BIPAP, and CPAP in patients with OHCA [38]. The additional use of PEEP (positive end-expiratory pressure) also seems to improve ventilation without negative effects on hemodynamics during CPR [39].

### Other ventilation strategies

There are some reports about the use of other conventional ventilation modes during resuscitation, such as pressure support ventilation (PSV) [40], passive ventilation with continuous positive airway pressure (CPAP) [41], or ultralow-tidal volume ventilation, in an experimental setting [42], but none of these ventilation modes has shown advantages and therefore has not become widely accepted.

### Ventilation with chest compression synchronized ventilation

**Fig. 4:**
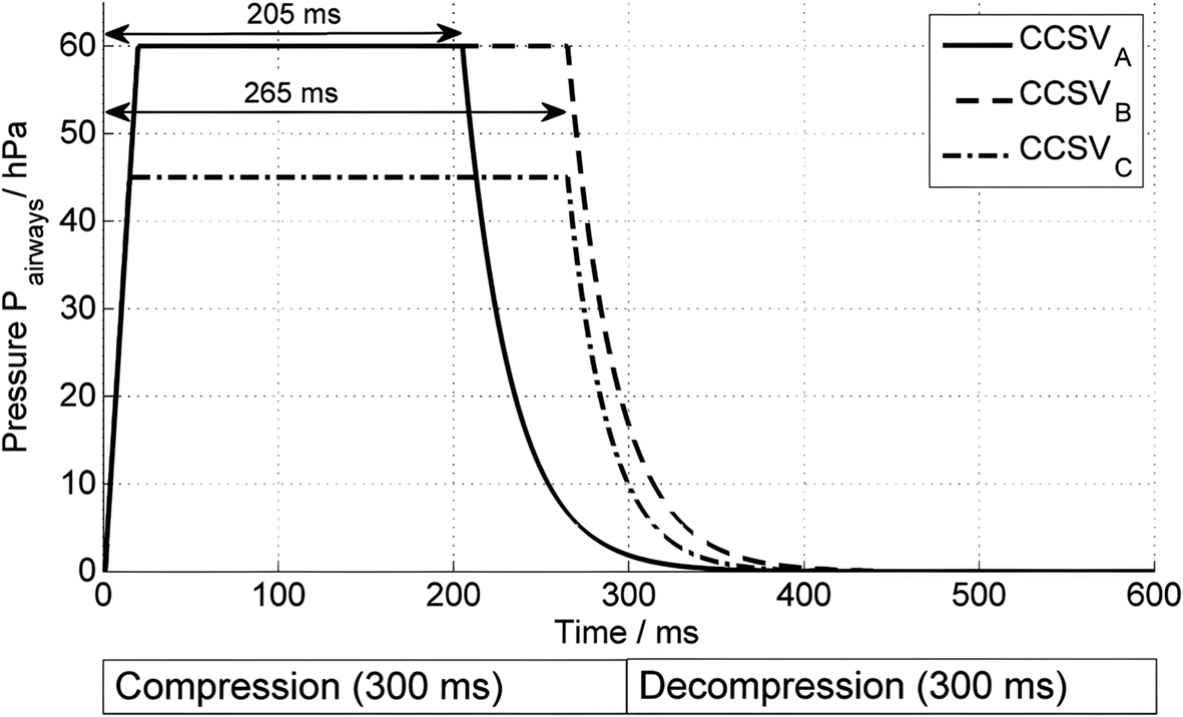
Pressure–time curves of the CCSV. Pressure–time curves of the three presets of chest compression-synchronized ventilation (CCSVA, CCSVB, and CCSVC) depending on the compression-decompression cycle. From Kill C., Galbas M. F., Neuhaus C., Hahn O., Wallot P., Kesper K., Wulf H., Dersch W. Chest compression synchronized ventilation versus intermitted positive pressure ventilation during cardiopulmonary resuscitation in a pig model. PLOSone 2015 26;10(5):e0127759

Since 2017, a new ventilation mode named chest compression synchronized ventilation (CCSV), which was designed exclusively for resuscitation, has been established and is available in a mobile ATV [43]. The key point in the development of such a ventilation mode was a special ventilation trigger. During CCSV, pressure delivery is triggered by expiratory gas flow caused by chest compression. This new trigger is activated by reaching three criteria at the same time. The first criterion is achieved when the airway pressure rises above a certain trigger level of 0.9–3.7 mbar above the PEEP. The second criterion is achieved when the airway pressure gradient reaches at least 25–375 mbar/s (i.e., when pressure rises fast enough), and the last criterion is met when at least 200–340 ms of expiration (chest decompression) has occurred. Synchronized with each chest compression, a short pressure-controlled inspiration with an inspiration time of approximately 0.2 s and a peak pressure of up to 60 mbar results in tidal volumes of 1–2 ml/kg body weight. An experimental investigation comparing different peak pressures and inspiration times showed the best results, with a peak inspiratory pressure of 60 mbar and an inspiratory time of 205 ms [44] (Fig. 4). The experimental data showed that ventilation with CCSV was superior to ventilation with volume-controlled ventilation mode IPPV or pressure-controlled ventilation mode BIPAP, providing normocarbia and higher oxygen partial pressures.

**Fig. 5:**
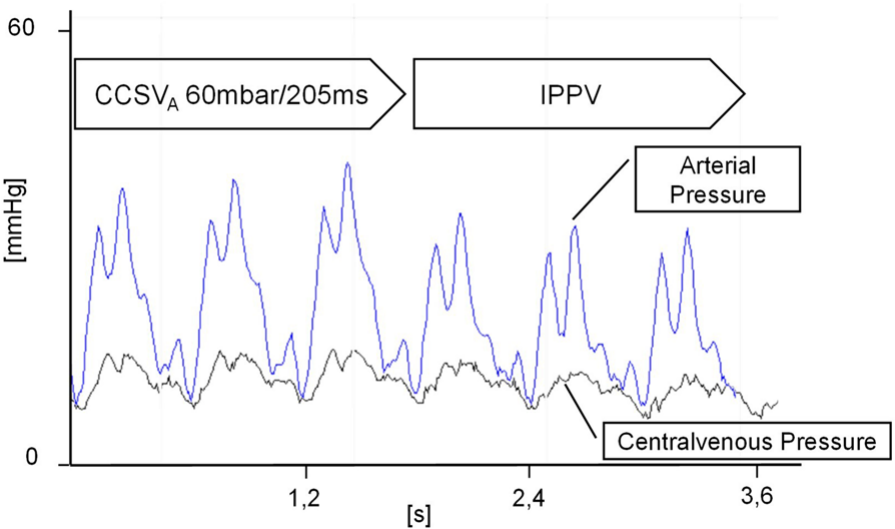
Influence of the CCSV versus the IPPV on arterial pressure (from Kill C., Galbas M., Neuhaus C., Hahn O., Wallot P., Kesper K., Wulf H., Dersch W. Chest compression synchronized ventilation versus intermitted positive pressure ventilation during cardiopulmonary resuscitation in a pig model*.* PLoS One. 2015 May 26;10(5):e0127759)

In addition, chest compression-synchronized insufflation led to an increase in the mean arterial pressure compared to that of the volume-controlled ventilation mode IPPV (Fig. 5). Recent experimental data have also shown that cerebral perfusion and oxygenation via the CCSV are superior to those via the IPPV [45]. Another experimental study in pigs showed that CCSV may have a positive impact on cytokine expression levels postresuscitation [46]. There are also the first reports on the prehospital use of CCSV [47], which also shows the advantages of CCSV in prolonged CPR [48]. Although there are several ongoing retrospectives, as well as experimental and prospective studies on CCSV, there are no published studies comparing CCSV with other ventilation modes in IHCA or OHCA patients.

### Future direction

The use of ventilation modes specifically developed for resuscitation, such as CCSV, should be applied more widely. Considering the altered pathophysiology of the lungs during resuscitation, more attention should be paid to the “pulmonary” aspect of ventilation during ALS in clinical research. It is essential to question established controlled and assisted ventilation modes from clinical practice, such as those used in intensive care medicine, and to investigate newer ventilation methods further clinically, such as CCSV ventilation, to substantiate their benefits for resuscitation.

Overall, the development of specialized ventilation modes for resuscitation should receive more support and be advanced through research activities in the field.

The use of EGA for ventilation during resuscitation appears to be disadvantageous, at least for prolonged durations of resuscitation, compared to ventilation through ETI.

Additionally, it remains crucial to scientifically address the question of which type of airway management, ETI or EGA, provides the greatest benefit for these specifically designed mechanical ventilation modes in the context of advanced airway management during resuscitation. This applies to both the OHCA and the IHCA scenarios.

Finding the best possible combination of optimal airway management and optimal ventilation mode during CPR should therefore be the future goal to achieve the best outcome from CPR.

## Conclusions

In addition to chest compressions, ventilation is still a key component of CPR. ETI is the most widely used method for airway management during CPR worldwide.

If available, mechanical ventilation should preferably be used, and manual ventilation should be avoided.

Chest compressions can adversely affect ventilation during CPR. Therefore, using standard mechanical ventilation modes (volume-controlled ventilation and pressure-controlled ventilation) during chest compressions may be disadvantageous. Today, with CCSV, there is a ventilation mode designed exclusively for resuscitation, which offers benefits such as improved oxygenation and blood pressure.

Finally, more research in the future should focus on ventilation during CPR to define the best ventilation strategies.

## Data Availability

Not applicable.

## Abbreviations

ALS: Advanced life support
ATV: Automated transport ventilator
BIPAP: Biphasic positive airway pressure
CPAP: Continuous positive airway pressure
CCSV: Chest compression synchronized ventilation
CPR: Cardiopulmonary resuscitation
eCPR: Extracorporeal cardiopulmonary resuscitation
EGA: Extraglottic airway device
EMS: Emergency medical service
ERC: European resuscitation council
ETI: Endotracheal intubation
ICU: Intensive care unit
IPPV: Intermittent positive pressure ventilation
IHCA: Inhospital cardiac arrest
OHCA: Out-of-hospital cardiac arrest
PEEP: Positive end-expiratory pressure
ROSC: Return of spontaneous circulation
SOP: Standard operating procedure

## Data availability

No datasets were generated or analysed during the current study.
